# Acute bone damage through liver-bone axis induced by thioacetamide in rats

**DOI:** 10.1186/s40360-022-00568-4

**Published:** 2022-05-07

**Authors:** Xiaoli Jin, Yang Li, Jianghua Li, Linyan Cheng, Yetao Yao, Hao Shen, Bili Wang, Jun Ren, Hang Ying, Jian Xu

**Affiliations:** 1 School of medical technology and information engineering, Zhejiang Chinese Medical University, Hangzhou, 310053 China; 2 FUDAN University, school of basic medical sciences, Shanghai, 200433 PR China; 3 Department of The Third Orthopaedic, The First Affiliated Hospital of Shihezi University School of Medicine Xinjiang Shihezi, Shihezi, 832008 China

**Keywords:** Bone damage, Hepatic fibrosis, Thioacetamide, Rat model

## Abstract

**Background:**

Thioacetamide (TAA) is used in various fields, such as synthetic drugs, organic chemical synthesis, and materials chemistry. TAA is mainly used to establish animal liver injury models and other organ damage models to explore their mechanisms for helping patients with liver disease. Liver damage can lead to abnormal expression of some enzymes in the serum, so we detected the appropriate enzyme levels in the serum of SD rats to verify the damage of TAA to the liver. More importantly, TAA caused bone damage is barely understood. Therefore, our research aims to establish a rat model reflecting the acute bone damage injury caused by TAA.

**Methods:**

The SD rats were intraperitoneally injected with normal saline (0.9%) or TAA (200 mg/kg, 400 mg/kg) for 1 month (once the other day). After the last intraperitoneal injection, serum samples from rats were used for biochemical tests. Masson staining is used to detect liver damage, and micro-CT is used to detect the changes in bone. Moreover, the three-point bending experiment was used to detect the force range of the hind limbs of SD rats.

**Results:**

Compared with the control group, after the intraperitoneal injection of TAA, the levels of aspartate aminotransferase (AST), alanine aminotransferase (ALT), uric acid (UA), total bile acid (TBA), alkaline phosphatase (ALP), carbamide (UREA) and creatinine (CREA) rose sharply, while the levels of serum content of total protein (TP), lactate dehydrogenase (LDH), calcium (Ca) and phosphorus (P) were severely reduced. After TAA administration, collagen fibers were deposited and liver fibrosis was obvious. Micro-CT results showed that the bone surface, tissue surface, bone volume, and tissue volume of rats with an intraperitoneal injection of TAA were significantly reduced. In addition, the bones of rats with an intraperitoneal injection of TAA can resist less pressure and are prone to fractures.

**Conclusions:**

TAA can cause liver damage in SD rats, which is explained by the changes in serum biochemical indicators and the deposition of liver collagen. More importantly, TAA can reduce bone mineral density and increase the separation of bone trabeculae in SD rats, and finally lead to bone injury. This suggests that TAA may become an ideal model to investigate abnormal bone metabolism after liver injury.

## Background

Thioacetamide (TAA) is a widely used commercial chemical, and it has been used as an organic solvent in textile and paper industries [1]. In 1948, Fitzhugh [2] found liver tumors in rats fed with TAA. The study exhibited a number of thiono-sulfur containing compounds, including TAA having toxic properties. These effects included bone marrow depression, liver damage, and lung damage [3]. In recent years, some researchers have paid attention to the reason TAA causes liver damage; TAA mediated by microsomal CYP2E1 to TAA-S or S-dioxide initiates cellular necrosis [4, 5]. Karantonis [6] suggested that reducing the levels of reactive oxygen species may improve liver damage, and the platelet-activating factor participated in the liver fibrotic process. Other researchers [7] also explored how to inhibit TAA toxicity in the liver, and how hepatic irradiation preconditioning enhances the effect of bone marrow-derived mesenchymal stem cells’ effect on TAA-induced liver fibrosis in rats. A study demonstrates that a small-molecule inhibitor of connexin 32 can protect against liver failure and death in wild-type mice when co-administered with TAA [8], as well as miR-34a-5p, a microRNA that was the most suitable and sensitive biomarker for TAA-treated hepatic carcinoma [9]. In addition, metabolic bone disease is common among patients with chronic liver disease [10, 11], associated with alterations in receptor activator of nuclear factor-kB ligand and osteoprotegerin serum levels [12]. When the function of the liver is abnormal, such as in chronic hepatitis C, liver fibrosis, liver cirrhosis and even liver cancer, the bone will also be damaged [13–15]. It is evident that TAA causes liver damage, although, TAA causing bone damage is barely understood. Bone damage associated with TAA toxicity was discovered as early as 1984. Lassila V [16] proposed that TAA-induced liver injury accompanied changes in serum proteins and alveolar bone, mainly around teeth, during occlusal stress and trauma, while significantly reducing the activity of osteoblasts and bone mass, and reducing formation of new bone. In 1996, Nakano [17] also proposed using carbon tetrachloride (CCL_4_) and TAA to establish liver cirrhosis models, as TAA and CCL_4_ induced liver cirrhosis can cause osteodystrophy, which is mainly responsible for bone volume reduction. The skeletal system includes bone, cartilages, ligaments, connective tissues, and the femur, a weight-bearing bone that transfers weight from the hip joint to the knee joint [18]. Our laboratory hopes to establish an animal model of osteoporosis by intraperitoneal injection of thioacetamide, and explore the mechanism of TAA causing bone damage.

## Methods

### Chemical

TAA was obtained from Sangon Biotech Co., Ltd. (Shanghai, China) , and > 98% purity was analyzed. It was dissolved in normal saline.

### Animals

All experiments in this study were conducted in accordance with the animal experiment guidelines of the Zhejiang Chinese Medical University Laboratory Animal Research Center (Approval No: IACUC-20181029-11). Sprague-Dawley rats (18 Male; 200–250 g) were obtained from Shanghai BK company and bred in the Animal Experimental Center of the Zhejiang Institute of Traditional Chinese Medicine in Hangzhou. The laboratory diet of the rats was based on a standard AIN-93 laboratory diet (Xietong Biotechnology, Nanjing, China) and kept in an ambient room with the following conditions: temperature (20 ± 2 °C), humidity 60% ∼ 65% and light (12 h light-dark cycle). They were acclimatized for 4 days before the study started. 18 rats were randomly divided into three groups(*n* = 6/group), (1) normal control, in which the rats received normal saline, the same injection volume with the heaviest in the high dose group, (2) low dose group, in which treated with TAA of 200 mg/Kg, (3) high dose group, in which treated with TAA of 400 mg/Kg. The experimental animals were injected intraperitoneally with TAA or normal saline every other day for 1 month, 15 times in total. Before rats were intraperitoneal injection, we had weighted every rat. Animal experiments were conducted according to the Guidelines for Animal Experimentation at our animal center. After fasting for 24 hours, all rats were anesthetized by intraperitoneal injection of sodium pentobarbital (45 mg / kg), and the retroorbital blood was collected for biochemical analysis. Then the abdominal cavity was then opened from the midline of the abdomen. Liver and limb bones were taken for follow-up experiments. The rats eventually died due to excessive blood loss in the whole process, and the rats did not feel severe pain.

### Serum biochemical analysis

Aspartate aminotransferase (AST), alanine aminotransferase (ALT), alkaline phosphatase (ALP), lactate dehydrogenase (LDH), total protein (TP), total bile acid (TBA) (AST、ALT、ALP、LDH、TP and TBA are indicators of liver function), calcium (Ca), phosphorus (P) (Ca and P are indicators of bone function), carbamide (UREA) and creatinine (CREA) uric acid (UA) (UREA、CREA and UA are indicators of renal function) were analyzed via Hitachi automatic analyzer 3100 (in the Zhejiang Chinese Medical University Laboratory Animal Research C enter) and present data.

### Histopathological examination

Immediately after sacrifice, the liver tissues were removed and instantly fixed in 10% neutral buffered formalin for 24 h. After fixation, the tissue samples were dehydrated with gradient ethanol from a low to a high concentration, dewaxed with xylene, soaked in wax, and then embedded to make tissue wax blocks by the biological tissue embedding machine (KeDee, China), which were all processed by the conventional paraffin embedding technique. Paraffin-embedded liver sections were cut to 4 μm thickness by the Paraffin slicer (Thermo, the USA), the wax strips were pl aced on the water surface of the biological tissue spread baking machine (KeDee, China), and scooped up with glass slides after leveled, which were baked through Masson’s trichrome (MT) stain and examined under a light microscope to detect collagen deposition. Masson staining showed that collagen fibers were blue and muscle fibers were red, indicating the number of threads in the tissue.

### Micro-computed tomography (μCT) analysis

The bone structure of rat left posterior femurs was determined by μCT scans (Bruker, Kontich, Belgium, Institute of Orthopaedic and Traumatology of Zhejiang Province, Hangzhou, Zhejiang, China), to assess the cortical bone and the trabecular bone, which was scanned with standard parameters (70 kV, 357 μA, power 25 W, exp. osure time 270 ms, resolution 18 μm). The intact femur metaphyseal regions were scanned by micro-CT and the range of interest (ROI) was about 2 mm from the femoral growth plate. Total surface area (TS), bone surface area (BS), bone volume (BV), total volume (TV), bone mineral density (BMD), structural model index (SMI), degree of anisotropy (DA), trabecular number (Tb.N), trabecular bone pattern factor (TBPf), trabecular separation/spacing (Tb.Sp), trabecular thickness (Tb.Th) of the femoral were determined.

### Three-point bending experiment

The three-point bending experiment was carried out at the Institute of Orthopedics and Traumatology of Zhejiang Chinese Medical University. The maximum load of rat femur was measured by the AGS-J series precision electronic universal material experiment machine. The femur was loaded onto the 17 mm span in an anterior-posterior position, and a load was applied to the midshaft of the femur, on the anterior surface, at a uniform speed of 10 mm/min until the bone specimen was broken. The load-deformation curve was plotted, and the analysis data was recorded.

### Statistical analysis

All experimental data are expressed as the means ± SD. Statistically significant differences between groups were assessed by a one-way analysis of variance (Anova) followed by Tukey’s post hoc test. All data were analyzed using the SPSS statistical package version 22.0 for Windows (IBM, Armonk, NY, USA). *P* < 0.05 indicated a statistically significant difference.

## Results

### TAA inhibited the growth of SD rats

**Fig. 1:**
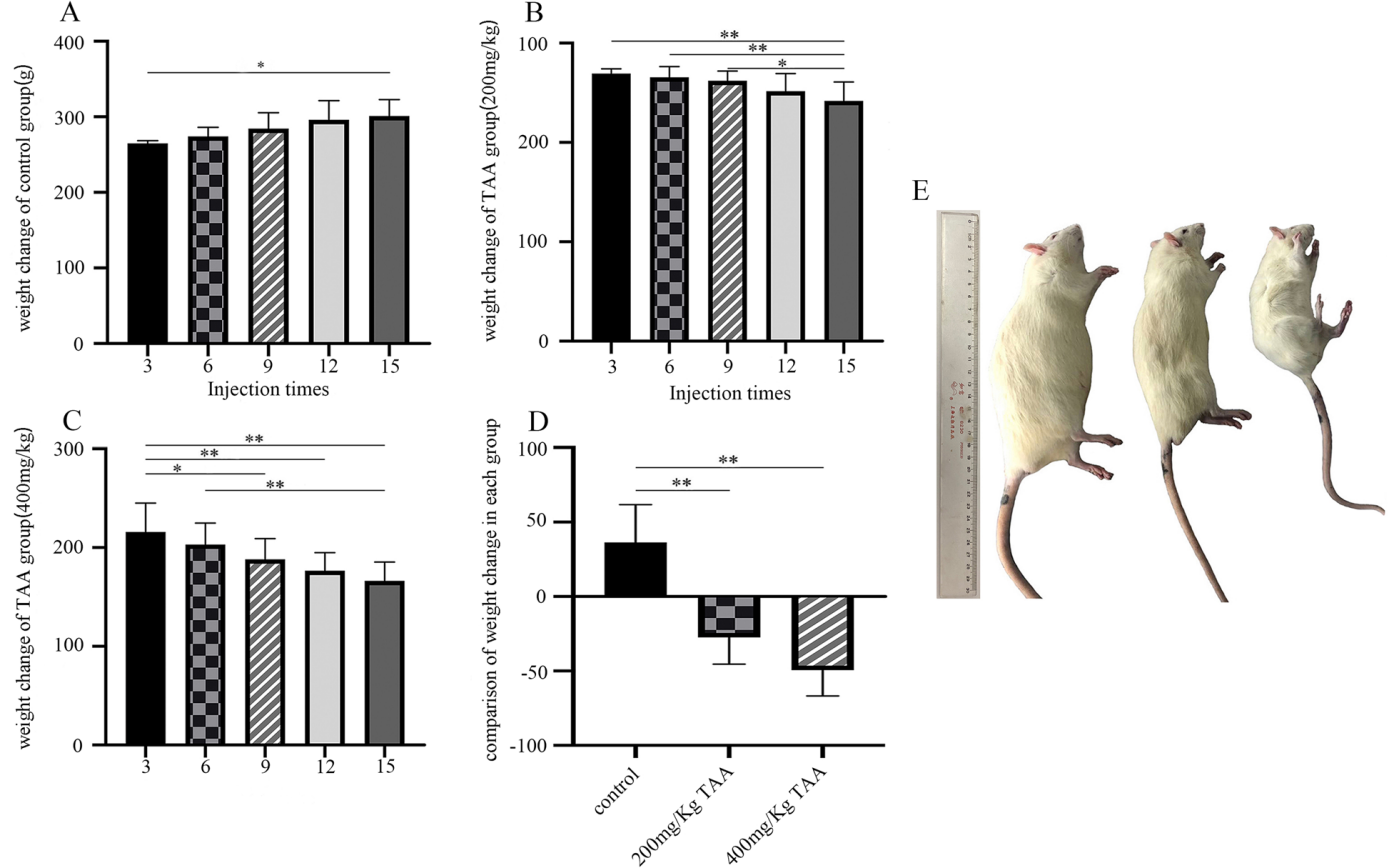
The effect of TAA on body weight of SD rats. **A**-**C** Body weight changes of SD rats in each group after intraperitoneal injection of TAA or normal saline for the third, sixth, ninth, twelfth and fifteenth times. **D** The weight of each group before the last intraperitoneal injection minus the weight before the first intraperitoneal injection was statistically analyzed, *P < 0.05, **P < 0.01. **E** Morphological comparison of SD rats in each group after intraperitoneal injection of TAA

After intraperitoneal injection of the drug, SD rats were anesthetized and sacrificed. It can be seen that TAA can inhibit the growth of SD rats (Fig.1). As the concentration of TAA increased, the growth and development inhibition of SD rats were more obvious.

### TAA induced liver injury

**Fig. 2:**
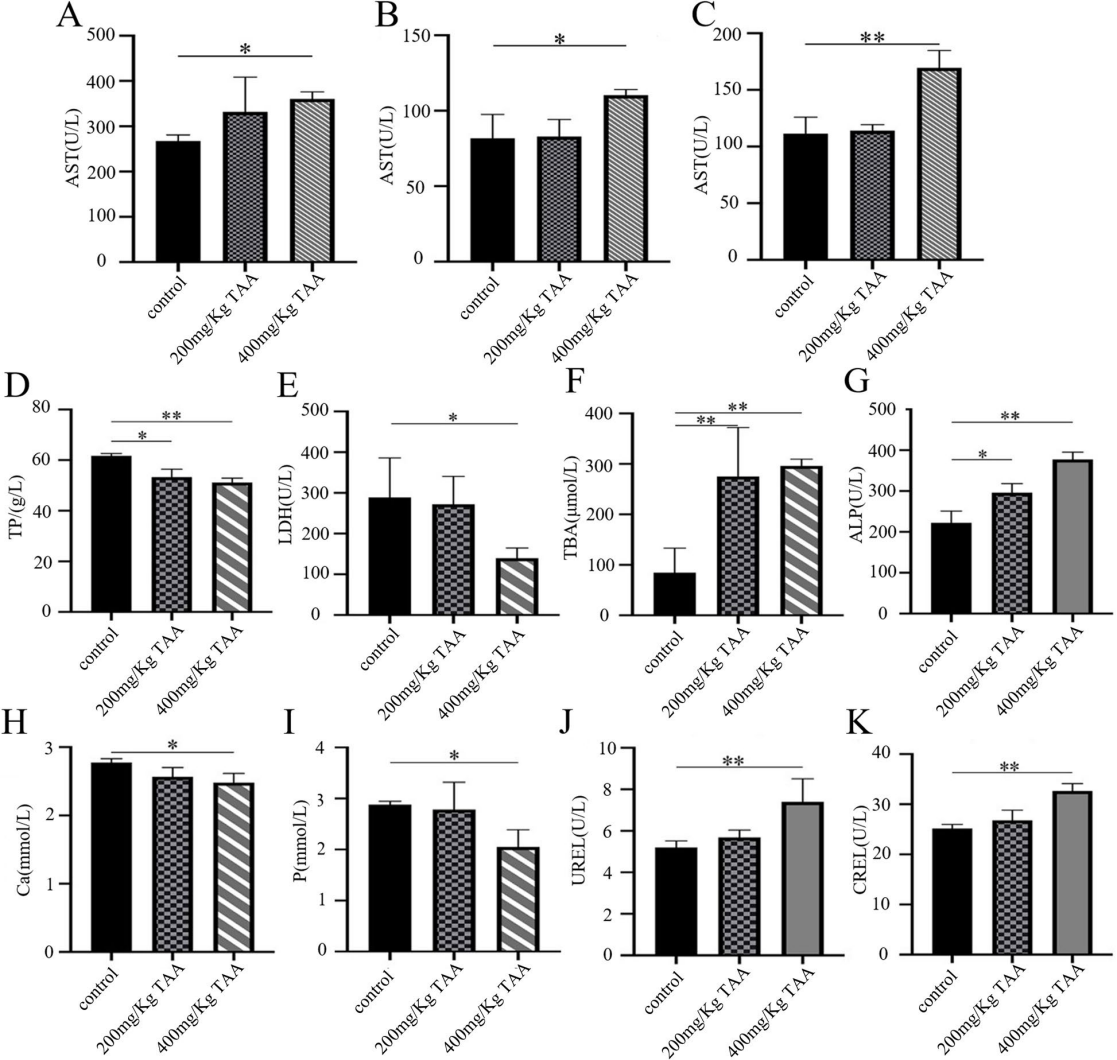
After intraperitoneal injection of TAA, the serum biochemical indexes of SD rats were changed. Contrast in **A** AST, **B** ALT, **C** UA, **D** TP, **E** LDH, **F** TBA, **G** ALP, **H** Ca, **I** P, **J** UREA and **K** CREA in each group, *P < 0.05, **P < 0.01

After the injection of normal saline and different doses of TAA, the relevant biochemical indicators changed significantly. The levels of serum AST, ALT, and UA were increased dramatically in the high dose group, while there was no obvious change in the low dose group. Figures 2D and E showed that after intraperitoneal injection of TAA, the serum TP and LDH of SD rats decreased, while TBA and ALP increased. It showed that TAA could cause liver damage. Compared with the normal control group, the levels of serum Ca and P decreased to varying degrees after intraperitoneal injection of TAA. However, the high dose group significantly increased UREA and CREA levels. This suggested that TAA may cause bone and kidney damage.

**Fig. 3:**
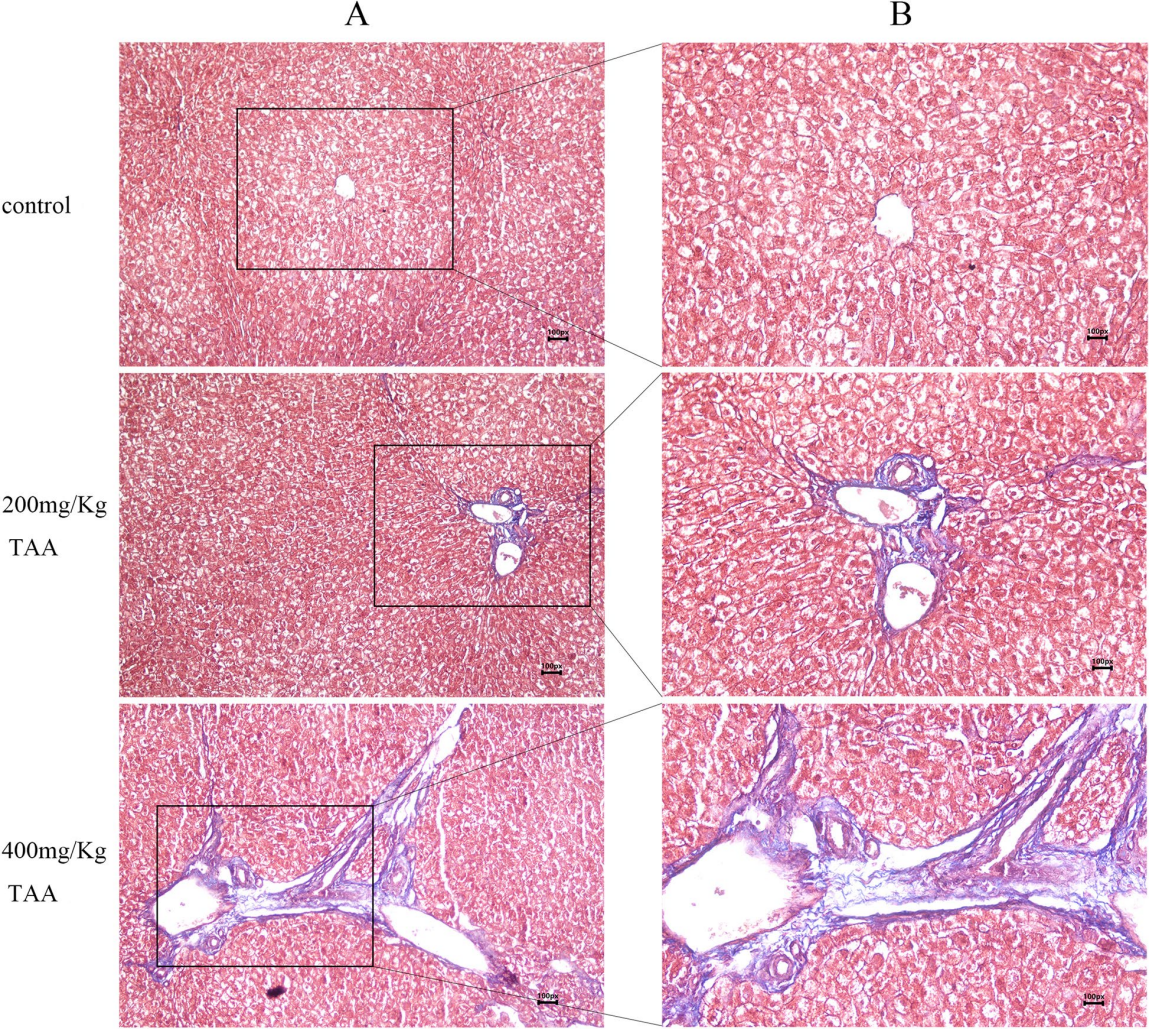
TAA induced liver collagen deposition in SD rats. **A**, **B** Masson staining pathology of the liver

**Fig. 4:**
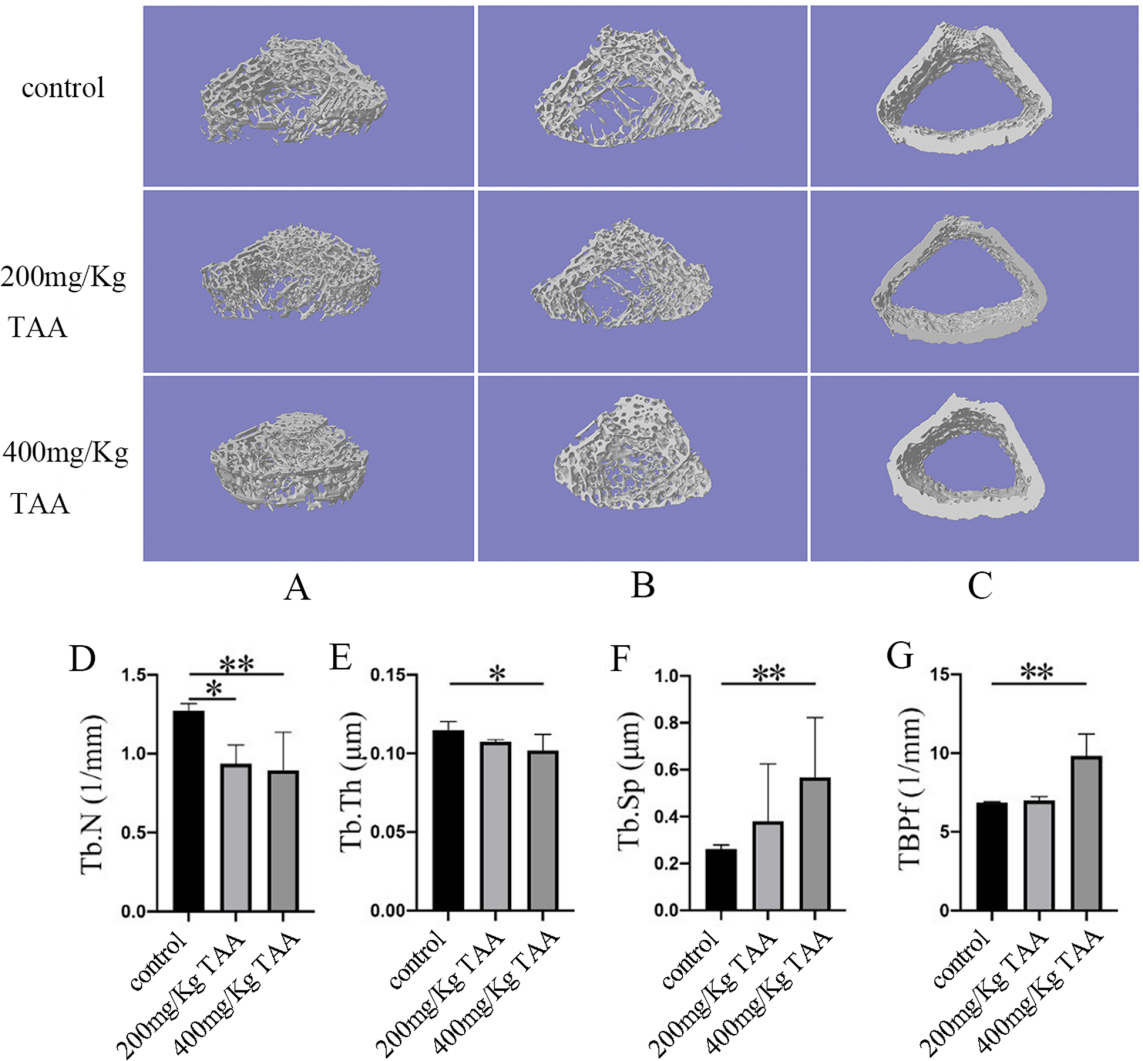
TAA induced bone loss in SD rats. **A**-**C** Radiographs of the longitudinal and transverse sections of the proximal femurs were obtained with a micro-CT apparatus. **D**-**G** The Tb. N, Tb. Th, Tb. Sp and TBPf of the femurs were determined by the micro-CT data, *P < 0.05, **P < 0.01

**Fig. 5:**
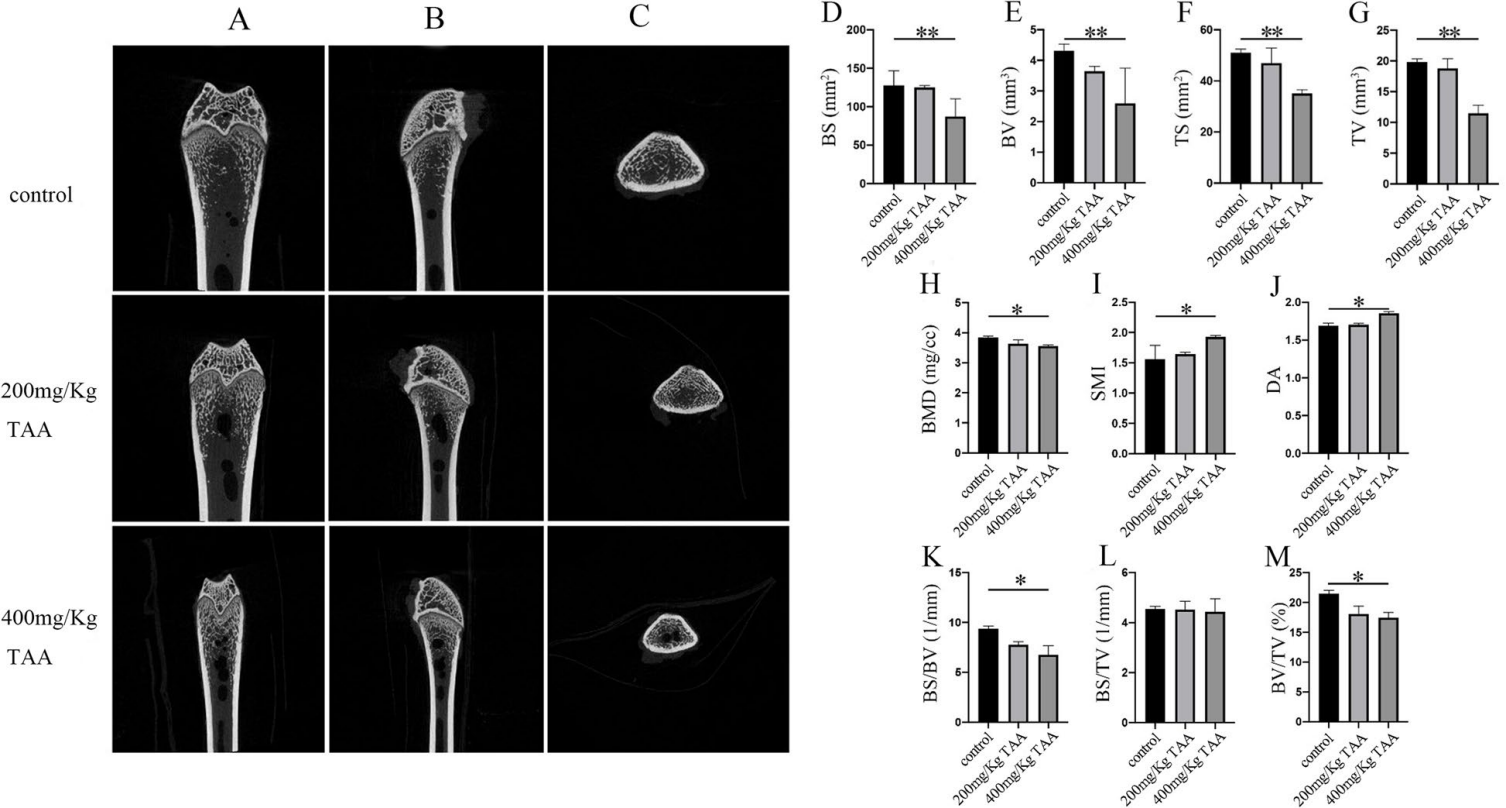
TAA induced bone loss in SD rats. Radiographs of the longitudinal and transverse sections of the proximal femurs were obtained with a micro-CT apparatus. **D**-**M** The BS, BV, TS, TV, BMD, SMI, DA, BS/BV, BS/TV and BV/TV of the femurs were determined by the micro-CT data, *P < 0.05, **P < 0.01

**Fig. 6:**
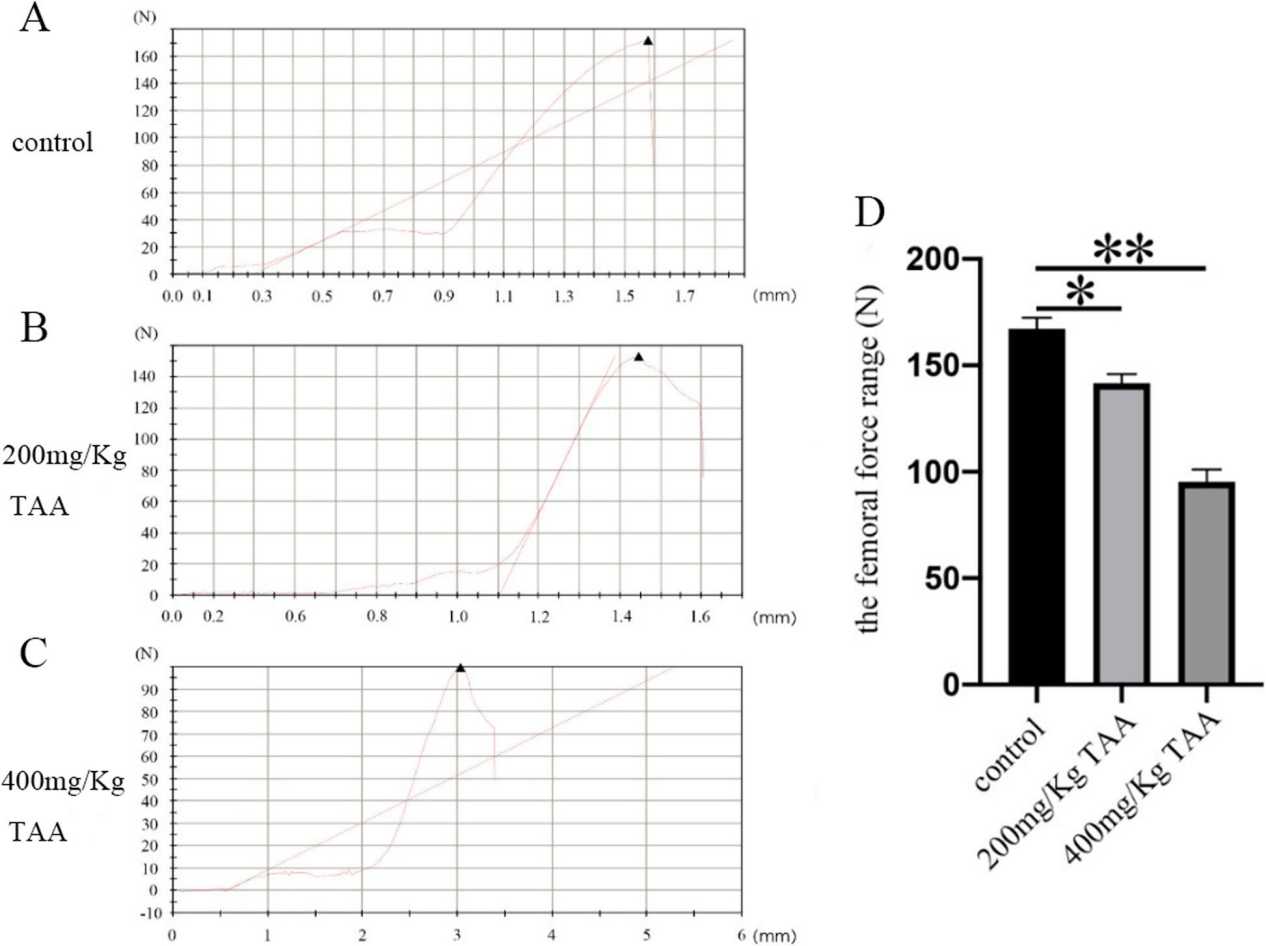
The effect of TAA on femoral load in SD rats. **A**-**C** Three-point bending test results of bone. **D** Statistical diagram of maximum load of each group, *P < 0.05, **P < 0.01

Figure 3 showed Masson staining of the liver-pathological sections of the control group and different doses of the TAA group. Masson staining showed that hepatocytes in the normal control group were closely connected without fibrous dysplasia. In the groups of intraperitoneal injection of TAA, there were many abnormal hyperplasias of reticular fibers in the portal area, and the fiber spacing widened, with obvious collagen deposition. This showed that TAA could induce liver fibrosis in SD rats.

### TAA induced bone injury in SD rats

The three-dimensional structure of the femur was analyzed by NRecon software (Fig. 4) to detect the possibility of bone injury in SD rats which was related to TAA treatment. At the same time, we obtained a planar Micro-CT image of the distal end (Fig. 5). It can clearly be concluded that from the normal control group to the TAA-treated group, the bone volume dropped dramatically, the thickness of trabecular bone decreased, and the separation of trabecular bone increased. At last, the three-point bending experiment was used to test the femoral force range of the hind limbs of SD rats (Fig. 6). The results showed that with the increase in TAA concentration, the force range of the femur decreased gradually.

## Discussion

In 1948, researchers suggested that TAA has hepatotoxicity, leading to liver fibrosis, and even producing liver tumors in a certain drug concentration and within a certain time. At the same time, the researchers could not find that TAA had an effect on other organs, and suggested that the extent to which certain organs were affected may not be discovered, and further research is needed [2]. Then, TAA has been shown to cause cholangiocarcinoma [19]. Some scholars have also found that TAA not only damages the liver of animals, but also affects the kidneys, brain, spleen, and bone of animals [16, 20–22]. When TAA treated the liver of the animal model the enzyme metabolism of the liver, protein, adipose, amino acid, and the messenger RNA was changed to varying degrees as compared to the normal group [9, 23–26]. However, most scholars are concerned about the role of TAA in the liver and its mechanism. A few scholars are concerned about the bone damage caused by TAA.

Our data underline that rats with severe liver diseases result in osteopenia, especially in weight-bearing bones. Changes in body weight levels were observed in the three groups, and significant weight loss was observed in 200 mg/kg TAA and 400 mg/kg TAA groups, indicating that intraperitoneal injection of TAA could affect the growth of rats. Additionally, the increased concentration of AST and ALT represented severe liver injury, and the increased concentration of UA, UREA, CREA, and TBA represented severe kidney injury, which correlates with other research [27, 28]. We also found a decreased concentration of TP and an increased concentration of ALP, which indicated liver disease [29]. The levels of rat serum ALT, AST, and ALP exposed to TAA were significantly increased, indicating that the liver cell membrane was impaired and their release into the bloodstream was increased after TAA administration. Our data showed serum calcium concentration of 200 mg/Kg TAA and 400 mg/Kg TAA group was decreased compared with the control group; the reason for decreased serum calcium is associated with the progression of cell injury since alterations in cell signaling play a determinant role in the toxicological processes [30]. Serum phosphorus is primarily in the form of inorganic phosphate, which is maintained within the physiological range by the regulation of bone formation, dietary absorption, renal excretion, and equilibration with intracellular stores. Long-standing phosphorus deficiency will increase the risk of osteomalacia [31]. The concentration of serum phosphorus in our data was decreased compared to the TAA-treated group with a control group, which is as expected in other literature. LDH is an insensitive index of all types of hepatic necrosis except hypoxic [32]; the level of LDH was not increased compared to the TAA-treated group with a control group in our studies. In our rat model, TAA-treated animals developed severe liver fibrosis as demonstrated by Masson staining, which correlates with other studies [33]. Masson staining showed that the deposition of liver collagen fibers increased after TAA administration, and liver fibrosis was obvious.

In conclusion, we successfully established a rat model of liver injury caused by TAA. Neal [3] has reported that thiono-sulfur compounds exhibited toxic properties in mammals, including bone marrow depression, and liver damage. Pauli Virtance considered increased osteoclastic resorption in the alveolar bone surrounding the occlusal stressed tooth in conjunction with the horizontal bone loss [34]. The μCT analysis of the bone revealed that the bone structure significantly changed in TAA-treated rats. The diameter of the femur was significantly reduced, indicating that the toxic effect of the drug affected the growth of bone in rats. BMD was unaffected in cortical bone. However, the most significant change observed in trabecular bone was the reduction of BV, TV, BS, and TS. Furthermore, a decrease in the diameter of the femur and the cortical bone thickness in the intraperitoneal TAA group was observed in Figs. 4 and 5, especially in the 400 mg/Kg TAA group compared with the control group. In the last, the force analysis of the femur represented a gradual down-trend from the normal group to the TAA-treated group. TAA administration was leading to bone fragility and increasing the risk of fracture. The μCT analysis of the bone revealed that the main part of skeletal injury caused by TAA was cortical bone, but the effect of TAA on trabecular bone was not so obvious. Therefore, our results suggest that TAA administration affects the on bone metabolism of SD rats, which may relate to liver injury, but no literature suggests that acute liver injury can quickly affect bone metabolism. In this rat model, significant changes in bone metabolism were observed after only 1 month, suggesting that it may be possible to quickly build a rat model to represent bone disease for studying the mechanism of liver damage.

The liver is a multifunctional organ that occupies a key position in the modulation of protein, lipid, and carbohydrate metabolism, and it also plays a significant role in mineral metabolism and growth [35]. It has been documented that bone loss is a primitive bone disease in patients with the early liver disease [36]. Therefore, if TAA is injected intraperitoneally, TAA or TAA complex may damage the bones of rats. Osteoblasts affected osteoclast formation, differentiation, and apoptosis through several pathways, such as OPG/RANKL/RANK, RANKL/LGR4/RANK, Ephrin2/ephB4, and Fas/FasL pathways [37]. Moschen AR demonstrated that the OPG/RANKL pathway is altered in patients with chronic liver disease, which regulates bone loss [12, 13]. In addition, rats with TAA-induced or carbon tetrachloride-induced cirrhosis showed a reduced bone volume and was histologically similar to human osteoporosis. Atsushi Nakano suggested that chronic parenchymal liver injury causes osteoporosis due to low bone formation rates and high resorption rates. The principal pathogenesis of HOD seems to be intestinal Ca malabsorption due to lower serum albumin and villous atrophy and serum levels of vitamin D metabolites have little influence on the pathogenesis of HOD [38]. However, in healthy postmenopausal women, vitamin D supplementation significantly improved hip bone density but did not significantly reduce hip fracture, while increasing the risk of kidney stones [14]. Although vitamin D had a key role in liver injury, its relationship with TAA-induced liver injury and bone disease required further research to clarify the potential link. Osteoporosis was associated with primary biliary cirrhosis (PBC), and was a risk factor for vertebral fracture [39]. It is necessary to further explore the relationship between TAA, PBC and vertebral fractures to determine their role in the pathogenesis of liver injury.

## Conclusions

Our experiment data suggest that TAA can damage the liver and even cause cancer as a widely used drug. It can also damage other organs, and when we choose a certain dose for intraperitoneal injection, it can also cause bone damage, which may pass through the liver bone axis. According to our experimental data, we suggest that TAA can cause severe damage to the weight-bearing bone of rats, especially the cortical bone and trabecular bone. The high dose of TAA caused the cortical bone to be significantly thinner; the ability to withstand huge external forces was significantly reduced, and the internal structure of the trabecular bone was seriously affected. In the end, we will continue to explore more in-depth pathological mechanisms between TAA, liver, and bone, and build a stable animal model to research TAA-caused bone disease.

## Data Availability

The datasets used and/or analyzed during the current study are available from the corresponding author on reasonable request.

## Abbreviations

TAA: Thioacetamide
AST: Aspartate aminotransferase
ALT: Alanine aminotransferase
UA: Uric acid
TBA: Total bile acid
ALP: Alkaline phosphatase
UREA: Carbamide
CREA: Creatinine
TP: Serum content of total protein
LDH: Lactate dehydrogenase
Ca: Calcium
P: Phosphorus
μCT: Micro-computed tomography
TS: Total surface area
BS: Bone surface area,
BV: Bone volume
TV: Total volume
BMD: Bone mineral density
SMI: Structural model index
DA: Degree of anisotropy
Tb.N: Trabecular number
TBPf: Trabecular bone pattern factor
Tb.Sp: Trabecular separation/spacing
Tb.Th: Trabecular thickness
